# *Clavibacter michiganensis* Downregulates Photosynthesis and Modifies Monolignols Metabolism Revealing a Crosstalk with Tomato Immune Responses

**DOI:** 10.3390/ijms22168442

**Published:** 2021-08-05

**Authors:** Dikran Tsitsekian, Gerasimos Daras, Konstantina Karamanou, Dimitris Templalexis, Konstantinos Koudounas, Dimitris Malliarakis, Theologos Koufakis, Dimitris Chatzopoulos, Dimitris Goumas, Vardis Ntoukakis, Polydefkis Hatzopoulos, Stamatis Rigas

**Affiliations:** 1 Laboratory of Molecular Biology, Department of Biotechnology, Agricultural University of Athens, Iera Odos 75, 11855 Athens, Greece; dtsitsekian@aua.gr (D.T.); gdaras@aua.gr (G.D.); kon.karamanou@gmail.com (K.K.); dimitempl@aua.gr (D.T.); koudounas@aua.gr (K.K.); 2 EA2106 Biomolécules et Biotechnologies Végétales, Université de Tours, 37200 Tours, France; 3 Laboratory of Plant Pathology-Bacteriology, Department of Agriculture, School of Agricultural Sciences, Hellenic Mediterranean University, Estavromenos, 71004 Heraklio, Greece; dim-mal@hotmail.com (D.M.); dgoumas@hmu.gr (D.G.); 4 AGRIS S.A., Imathia Horticulture Center, 59300 Kleidi, Greece; koufakis@agris.gr; 5 Biomedical Research Foundation of the Academy of Athens, 11527 Athens, Greece; dimitrischat@gmail.com; 6 School of Life Sciences and Warwick Integrative Synthetic Biology Centre, University of Warwick, Coventry CV4 7AL, UK; V.Ntoukakis@warwick.ac.uk

**Keywords:** *Solanum lycopersicum*, gram-positive, transcriptomics, RNA-seq, photosynthesis, plant pathogen interaction, phenylpropanoids, monolignols, defense lignin, plant immunity

## Abstract

The gram-positive pathogenic bacterium *Clavibacter michiganensis* subsp. *michiganensis* (*Cmm*) causes bacterial canker disease in tomato, affecting crop yield and fruit quality. To understand how tomato plants respond, the dynamic expression profile of host genes was analyzed upon *Cmm* infection. Symptoms of bacterial canker became evident from the third day. As the disease progressed, the bacterial population increased in planta, reaching the highest level at six days and remained constant till the twelfth day post inoculation. These two time points were selected for transcriptomics. A progressive down-regulation of key genes encoding for components of the photosynthetic apparatus was observed. Two temporally separated defense responses were observed, which were to an extent interdependent. During the primary response, genes of the phenylpropanoid pathway were diverted towards the synthesis of monolignols away from S-lignin. In dicots, lignin polymers mainly consist of G- and S-units, playing an important role in defense. The twist towards G-lignin enrichment is consistent with previous findings, highlighting a response to generate an early protective barrier and to achieve a tight interplay between lignin recomposition and the primary defense response mechanism. Upon progression of *Cmm* infection, the temporal deactivation of phenylpropanoids coincided with the upregulation of genes that belong in a secondary response mechanism, supporting an elegant reprogramming of the host transcriptome to establish a robust defense apparatus and suppress pathogen invasion. This high-throughput analysis reveals a dynamic reorganization of plant defense mechanisms upon bacterial infection to implement an array of barriers preventing pathogen invasion and spread.

## 1. Introduction

Tomato (*Solanum lycopersicum*) is an important vegetable of high economic value due to the taste, high yield and high content of beneficial to human health compounds [1]. However, tomato plants are susceptible to various phytopathological agents of bacterial origin that compromise the yield and quality of the product. Bacterial speck disease, caused by *Pseudomonas syringae* pv. *tomato* (*Pst*), thrives in cool and moist environments causing severe growth defects [2]. *Pst* mostly affects the stems and leaves and is recognized by small, dark spots surrounded by a yellow ring. Bacterial spot affects the stems, leaves and fruits particularly in wet and humid environments and causes loss of leaves due to various *Xanthomonas* species [3]. Bacterial wilt is induced by the soil-borne bacterium *Ralstonia solanacearum* and results in leaf chlorosis leading to leaf loss. Eventually, the entire plant suffers wilting [3]. 

While most of the bacterial diseases of tomato are caused by gram-negative bacteria, *Clavibacter michiganensis* subsp. *michiganensis* (*Cmm*) the causal agent of bacterial canker is a gram-positive pathogen [4]. As a systematic vascular disease, bacterial canker affects all parts of tomato plants causing browning and wilting. *Cmm* was initially isolated in 1909 in Michigan and rapidly spread worldwide [5,6], frequently leading to outbreaks [7,8]. The worldwide spread of this bacterium is facilitated by contaminated seed stocks, in which a single infected seed in 10,000 is capable of initiating an epidemic [6,9]. Hence, *Cmm* has been added to the list of global quarantine microorganisms [9]. To date, there is no report of any resistant cultivar and effective chemical control of *Cmm* is still scarce [10]. 

The virulence of *Cmm* is attributed to numerous putative serine proteases and cell-wall-degrading enzymes, which are encoded by genes localized on a chromosomal pathogenicity locus and two plasmids (pCM1 and pCM2) [11,12]. *Cmm* can enter the tomato epiphytically through natural openings and wounds or spread from infected seeds [6,9,13]. Once inside a plant, the bacterium multiplies in xylem vessels, forming extensive biofilm-like structures, assisting pathogen colonization and movement [14]. Systemic infection with high in planta bacterial populations leads to typical wilting, stem canker and vascular discoloration [6,15]. Intriguingly, the pathogen leaks from cankers and, in combination with rain and wind, can spread, infecting distal leaves, fruits, and surrounding plants [6,16]. Bacteria present on the fruit surface can cause “bird’s-eye” lesions that consist of small tan dots with white halos [6,17]. 

Plant immunity relies on a two-tiered innate plant immune system. Initially, microbe-associated molecular patterns (MAMP) are detected by host plasma membrane-localized receptors and activate MAMP-triggered immunity (MTI). However, adapted pathogens can cause disease primarily by employing effector proteins capable of attenuating MTI. In turn, plants have evolved intracellular receptors capable to recognize pathogen-derived effectors and initiate effector-triggered immunity (ETI) [18,19,20,21,22,23]. In contrast to the gram-negative *Pseudomonas*, *Xanthomonas* and *Ralstonia* that utilize a type 3 secretion system (T3SS) to translocate effectors into the plant cells, the gram-positive *Cmm* doesn’t encode a T3SS and therefore, a typical ETI is ambiguous in the *Cmm*-host interaction [11,24]. 

Bacterial diseases, including bacterial canker, are expected to become more aggressive in the near future due to climate instability with devastating effects on basic food-producing areas [25]. Models predict that dynamic climate changes will promote plant disease dispersal, pathogen overwintering, emergence of new adapted pathogenic strains and decreased crop marketability [25]. Understanding how tomato responds to a bacterial threat is a challenging task to decipher its molecular mechanisms of defense and generate disease-resistant cultivars.

The tomato genome release [26] together with the pan-genome analysis of 725 tomato accessions [27] commenced holistic approaches of next-generation sequencing (NGS) capabilities allowing the profiling of genome-wide gene expression levels. These NGS-based RNA sequencing (RNA-Seq) approaches have become valuable tools for early diagnosis and new disease management strategies aiming to reduce pathogen dispersal, reduce crop loss and diminish the agrochemical footprint on the environment. 

By applying an RNA-seq analysis of *Cmm* infected tomato plants, we investigated the transcriptome of tomato during the progress of bacterial infection. This led to the identification of differentially expressed genes (DEGs) that helped us to construct an atlas of the most affected plant biological processes. Functional analysis of tomato DEGs revealed three main biological processes altered by *Cmm* infection, namely photosynthesis, plant-pathogen interaction and phenylpropanoid metabolism. Given that *Cmm* is a virulent bacterial pathogen in tomato, our results revealed a number of highly *Cmm*-responsive tomato genes during disease development. Moreover, the transcriptome atlas provides evidence about a tight interplay between monolignols biosynthesis and mechanisms of plant defense with practical implications towards breeding of disease-resistant tomato plants.

## 2. Results

### 2.1. Cmm Infection Dynamics

**Figure 1:**
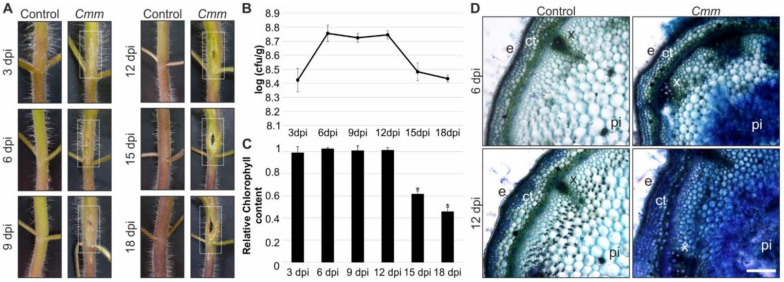
Disease symptoms in tomato plants infected with *Cmm*. (**A**) Bacterial canker lesions on inflorescence stems at 3, 6, 9, 12, 15 and 18 dpi. The dashed boxes in white indicate the symptoms. (**B**) Bacterial growth in tomato stem samples, which were harvested at different time points during an 18-day period after inoculation. Data represent the mean ± SD (n = 4). (**C**) Effect of *Cmm* infection on total chlorophylls of the true leaves. The relative chlorophyll content was calculated by comparing the concentration of chlorophylls quantified in mg/g of fresh weight between *Cmm* infected and control plants. Data are shown as means ± SD (n = 3). Asterisks indicate significant differences (*t*-test) between *Cmm* infected and control plants (*p* ≤ 0.05). (**D**) Trypan blue stained cross-sections at 6 and 12 dpi stems of tomato plants; e: epidermis, ct: cortex, x: xylem, pi: pith. (Scale bar = 500 μm).

An experimental system was developed to achieve a reproducible infection protocol of tomato plants by *Cmm* (Supplementary Figure S1). Tomato hybrid line Ekstasis F1 plants were inoculated by injecting a suspension culture of *Cmm* bacteria into the stem region between cotyledons and the plants were monitored daily for canker disease development. Canker lesions became visible at the inoculation site of the stem 3 days post infection (dpi) and were profoundly pronounced from 12 dpi (Figure 1A). Τo quantify the bacterial population inside the plant body after the infection, the bacterial growth was assessed for a period of 18 days, beginning from 3 dpi (Figure 1B). Bacterial growth in planta reached a maximum at 6 dpi, which was maintained until 12 dpi and then the bacterial population gradually reduced to the initial levels of 3 dpi (Figure 1B).

Even though the bacterial population progressively increased, the symptoms became acute beyond 12 dpi generating a trauma at the site of inoculation (Figure 1A). At 18dpi, the bacterial infection caused severe dysmorphia of the infected stems with devastating effects on major biological processes. As photosynthesis is a process indicative of plant health and growth, the effect of the bacterial infection was determined. The levels of total chlorophylls in the leaves of the infected plants were similar compared to the control plants up to 12 dpi. However, following the growth curve of *Cmm* bacteria in planta, a significant decrease of the photosynthetic pigments was detected in the true leaves of 15 dpi infected tomato plants (Figure 1C). Given that a drastic distortion of stem tissue morphology appeared at 18 dpi (Figure 1A), chlorophylls reached a low level in the true leaves of the infected plants showing a reduction of approximately 50% compared to the control (Figure 1C). 

The analysis of chlorophylls demonstrated that the bacterial infection, which initially caused destruction of the stems, finally resulted in leaf distortion. In addition, the staining pattern of stem sections over the site of bacterial infection with trypan blue dye was obvious at 6 dpi and became denser at 12 dpi indicative of the cell death effect originating from the progression of the disease (Figure 1D). Taken these results together, our experimental system of *Cmm* infection was efficient causing proximal and distal symptoms. Stem canker was evident as a proximal to the site of infection symptom, promoting cell death. In the distal leaves, *Cmm* affected photosynthesis associated with carbon fixation that drives plant growth.

### 2.2. Tomato Transcriptional Response to Cmm Revealed a Major Induction of Gene Expression

To gain further insights into the molecular mechanisms controlling tomato defense response upon *Cmm* infection, transcriptome analysis of the infected plants was performed. As the population of *Cmm* in planta remained constantly high and virulent within the period of 6 and 12 dpi (Figure 1A,B), RNA was isolated from stem sections spanning 1 cm over the infection site of these two time points. Deep sequencing of the transcriptome was performed in triplicates. RNA-sequencing (RNA-seq) data analysis resulted in 135M clean reads on average, with a mapping ratio of approximately 94% between all twelve samples demonstrating an efficient coverage of the tomato genome (Supplementary Table S1). Given that the tomato genome encompasses 34,075 genes, the total genes identified by our analysis were approximately 23,200 for the control and 23,600 for *Cmm* infected plants, which corresponded to 33,000 and 33,600 transcripts, respectively (Supplementary Table S2). The results were highly reproducible within each RNA-seq sample revealing a deep and satisfactory representation of the tomato transcriptome.

**Figure 2:**
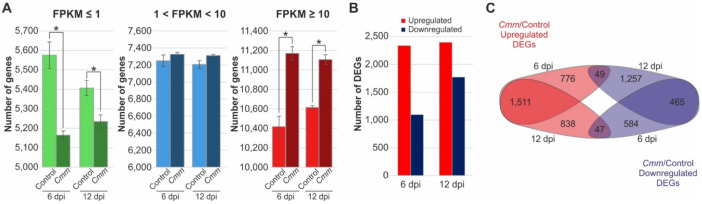
Overview of differentially expressed genes (DEGs) upon *Cmm* infection at 6 and 12 dpi. (**A**) Comparative analysis of gene expression distribution among the samples of control and *Cmm* infected plants. Red bars depict the highly expressed genes (FPKM ≥ 10), blue bars the genes with intermediate level of expression (1 < FPKM < 10), while green bars depict genes with low expression levels (FPKM ≤ 1). Values are mean ± standard deviation of three biological replicates of each sample. * *p* < 0.05 indicates significant difference between control and *Cmm* infected samples judged by Student’s *t*-test. (**B**) The number of genes that were significantly upregulated or downregulated in response to *Cmm* infection at 6 and 12 dpi. (**C**) Venn diagram showing a pairwise comparison of the number of upregulated and downregulated genes at 6 and 12 dpi of *Cmm* infection. *Padj* < 0.05 and log_2_FC ≥ 1 or ≤ −1.

To screen for DEGs, the parameters of adjusted *p*-value (*Padj*) and fold change (log_2_FC) were set to *Padj* < 0.05 and log_2_FC ≥ 1 or ≤−1, as criteria to select genes with a significant two-fold change. Gene expression distribution among the samples revealed a prominent overrepresentation of highly expressed genes (FPKM ≥ 10) contrary to an underrepresentation of low expressed genes (FPKM ≤ 1) in *Cmm* infected plants (Figure 2A). By comparing the number of genes, a clear and strong induction was evident regarding the group of genes characterized by high levels of expression in response to *Cmm* infection compared to the control plants at 6- and 12-days post infection. On the contrary, a great reduction of genes numbers that showed low expression levels at both 6 and 12 dpi upon *Cmm* infection, was observed. This distribution pattern of gene expression level was consistent between all three replicates of each experiment demonstrating that the results were highly reproducible and robust (Supplementary Figure S2). Interestingly, a correlation analysis of the samples revealed a strong relationship among the 6 and 12 dpi *Cmm* infected samples, contrary to the control samples at 6 and 12 dpi due to the growth of plants within the 6-day period (Supplementary Figure S3). Based on these observations, it is reasonable to suggest that *Cmm* infection resulted in a dynamic and coordinated reprogramming of the tomato transcriptome by changing the equilibrium of genes with low or high expression under non-infected conditions.

In line with this notion, analysis of the DEGs between infected and control plants at the two time points was performed. The analysis revealed 3432 DEGs at 6 dpi and 4167 at 12 dpi, corresponding to approximately 10% and 12% of the tomato genome coding genes, respectively (Figure 2B). These DEGs were further categorized into three groups (Figure 2C). The first group included 3125 genes that were upregulated upon *Cmm* infection, which were subdivided into 776 and 838 DEGs that were solely induced at 6 and 12 dpi, respectively, while 1511 genes were commonly induced at both time points (Figure 2C). The second group consisted of 2306 downregulated genes of which 584 showed reduced expression at 6 dpi and 1257 at 12 dpi, whereas 465 were presented at both time points and downregulated (Figure 2C). A distinct third category included 49 and 47 genes characterized by opposite expression responses upon comparison with DEGs of 6 and 12 dpi most likely being genes affected by not only the infection (Figure 2C). To acquire a general overview of transcriptome changes, a gene ontology (GO) analysis of DEGs at both time points of *Cmm* infection was performed (Supplementary Figure S4). The gene set enrichment analysis demonstrated common responses associated with terms such as “response to biotic stimulus”, “photosynthesis,” “phenylpropanoid metabolism,” “lignin metabolism” and “defense response”. Taken together, these results, had in total 5527 DEGs detected at both time points of *Cmm* infection, the majority of which were induced reflecting the dynamic response of the tomato transcriptome. 

### 2.3. Cmm Infection Highly Induced Host Genes Associated with Plant Defense and Disease Resistance

**Figure 3:**
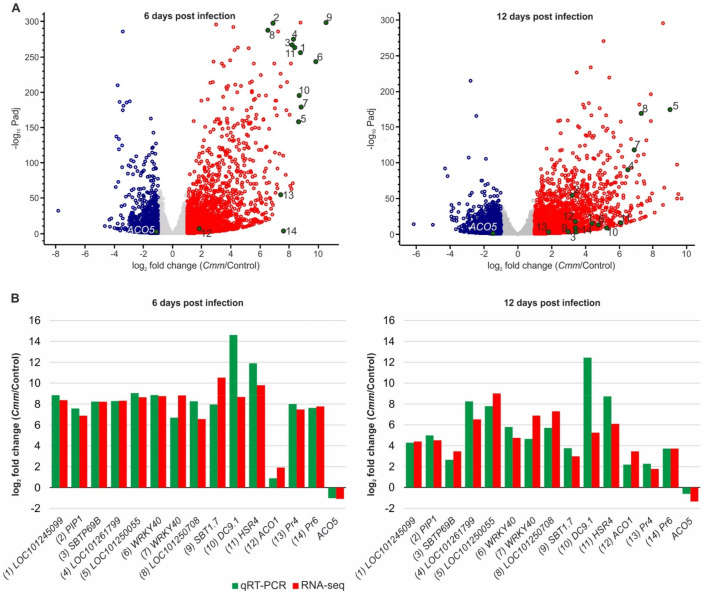
Differentially expressed genes of *Cmm* infected tomato stems. (**A**) Volcano plots of differentially expressed genes (DEGs) identified by RNA-seq analysis of *Cmm* infected versus control plants at 6 (left panel) and 12 dpi (right panel). Red and blue dots indicate up- and down- regulated genes, respectively. Green dots with numbers indicate genes selected for qRT-PCR validation of the transcriptome analysis results. (**B**) Comparative analysis of gene transcription results derived from qRT-PCR and RNA-seq transcriptome analysis. Green bars represent the fold change value calculated by qRT-PCR analysis as the log2 ratio between the quantitative median expression of the gene in *Cmm* infected plants relative to the control (n = 4). Red bars represent the fold change value expressed as the log2 between the ratio of the mean FPKM gene expression in *Cmm* infected and control plants obtained by RNA-seq analysis (n = 3). Positive values correlate with upregulated gene expression, whereas negative values with downregulation of gene (*ACO5*) expression.

As the expression of most *Cmm*-responsive genes was upregulated, a subset of the highly induced host genes was resolved, based on the level of expression at 6 dpi compared to the control plants (log_2_FC ≥ 6; Figure 3A, Supplementary Table S3). Prominent in the category of *Cmm*-responsive genes classified molecular components involved in protein quality control and particularly protein degradation including subtilisin-like serine proteases *SBTP69B* (spot #3) and *SBT1.7* (spot #9) and *PIP1* (spot #2). There were also two genes encoding members of the *WRKY* family of transcription factors (spots #6 and #7) and *Hyper-Sensitivity-Related 4* (*HSR4*; spot #11) that were highly expressed at 6 dpi (Figure 3A). These genes encode pathogenesis-related proteins and were highly induced at 6 dpi, whereas their expression was reduced at 12 dpi. Notably, among the *Cmm*-modulated tomato genes also classified, *1-aminocyclopropane-1-carboxylic acid* (*ACC*) *oxidase1* (*ACO1*; spot #12), *Pathogenesis-related proteins Pr4* (spot #13) and *Pr6* (spot #14), which are related to ethylene synthesis and response [28]. In tomato, at least five members of the *ACO* family regulate ethylene biosynthesis but show different expression patterns in response to biotic cues. This is evident especially for *ACO5*, which contrary to *ACO1*, showed downregulation of gene expression at both of the analyzed time points upon *Cmm* infection. Interestingly, our tomato transcriptome analysis in response to *Cmm* identified an identical cluster of highly expressed genes in comparison to a microarray approach validating the biological significance of our findings [28].

To further validate the accuracy and repeatability of RNA-seq data, quantitative real-time polymerase chain reaction (qRT-PCR) analysis was performed. Initially, at 6 dpi, the expression pattern of all selected genes was similar between the two experimental approaches (Supplementary Figure S5). This was further confirmed by calculating the fold change of gene expression using a log2 scale, which again supported the reproducibility of RNA-seq data compared to the experimental validation by qRT-PCR (Figure 3B). While the level of expression of many of these genes was reduced at 12 dpi compared to 6 dpi (Figure 3A, Supplementary Table S3), no significant differences were identified when comparing the two experimental approaches (Figure 3B, Supplementary Figure S5). The quantitative analysis of gene expression confirmed the downregulated pattern of *ACO5* at both time points, contrary to the rest of the genes. Together, these results indicate a strong and satisfactory correlation of qRT-PCR data with the outcome of transcriptome analysis, confirming the pattern of DEGs identified by RNA-seq analysis at both time points of tomato infection by *Cmm*.

### 2.4. Classification of Cmm Responsive Genes in Terms of Gene Expression Pattern and Biological Function

**Figure 4:**
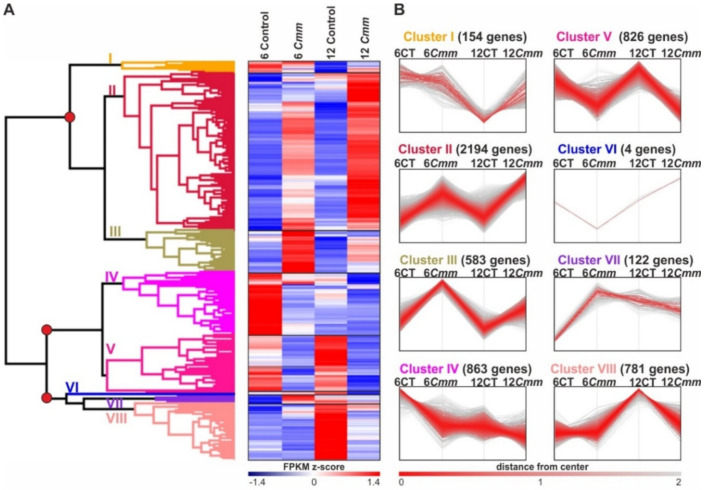
Sorting of tomato DEGs upon *Cmm* infection. (**A**) Hierarchical clustering and heat map of the 5527 DEGs showing the expression pattern of *Cmm* responsive genes at both time points. The red circles designate the nodes of the three major groups: the first group includes Clusters I–III of the upregulated DEGs, the second group Clusters IV–V of the downregulated DEGs and the third group Clusters VI–VII of DEGs without a distinctive pattern of expression between the two time points. (**B**) Expression pattern of the 5527 DEGs within the eight clusters. The y-axis represents the level of gene expression, whereas the x-axis represents the type of samples. Each line represents the pattern of expression of an individual gene within each Cluster. Red to gray scaling shows the distance of each gene from the center of the Cluster according to *k*-means clustering, in terms of gene expression pattern. In parenthesis the gene number of each cluster. CT: control plants and *Cmm*: *Cmm* infected plants.

To explore the host cellular processes affected by *Cmm* infection, further investigation of DEGs was performed. A hierarchical clustering of all 5527 DEGs revealed that they can be categorized into three distinct groups (Figure 4A). The first group was the largest with 2931 genes that showed a clear induction of expression upon *Cmm* infection. The genes of this group were subsequently categorized into three Clusters I-III, of which Clusters II and III containing 2194 and 583 genes, respectively, were highly induced at both 6 and 12 dpi (Figure 4B). The second group contained 1689 genes and was further subdivided into two clusters (IV-V, Figure 4A). The 826 genes of Cluster V showed a prominent downregulation upon *Cmm* infection at both 6 and 12 dpi (Figure 4B). The third group comprising from Clusters VI-VIII, contained 907 genes that did not display any apparent pattern between the two time points (Figure 4A,B). To exclude genes that display differential expression due to developmental differences between 6 and 12 dpi, we chose to further analyze the DEGs that showed a clear up- or down- regulation on both the time points examined, namely Clusters II, III and V.

**Figure 5:**
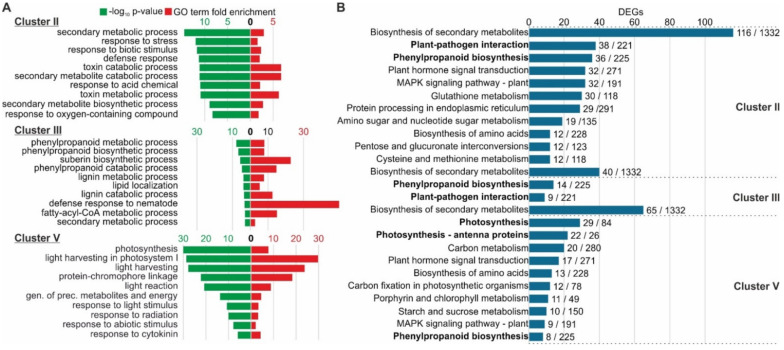
Functional classification of *Cmm* responsive genes within Clusters II, III and V. (**A**) Gene ontology (GO) analysis of differentially enriched tomato genes. (**B**) Classification into functional KEGG pathways of DEGs. The fractions on the right display the number of DEGs identified to the total number of genes of each pathway.

Gene Ontology (GO) enrichment analysis was performed for the 3603 DEGs included in Clusters II, III and V. In particular, Clusters II and III predominantly including the upregulated DEGs upon *Cmm* infection, were significantly enriched in biological processes mainly associated with the secondary metabolism, stress response including response to biotic stimulus, defense response and the phenylpropanoid biosynthetic pathway (Figure 5A). Notably, Cluster III was additionally enriched in terms of lignin and suberin metabolism that were significantly overrepresented (Figure 5A). The downregulated DEGs were represented in Cluster V. Hence, Cluster V constitutes a unique group of DEGs indicating that photosynthesis was the most dramatically affected process and especially light harvesting in photosystem I and reaction to light (Figure 5A).

Complementary to GO enrichment analysis, a Kyoto Encyclopedia of Genes and Genomes (KEGG) pathways enrichment analysis was performed to assess with confidence the biological functions and interactions of DEGs due to *Cmm* infection. The main KEGG pathway affected in these three Clusters was secondary metabolism. In Cluster II, however, which includes a significant number of induced DEGs, the main activated pathways were involved in plant-pathogen interaction, phenylpropanoid biosynthesis, plant hormone signaling and MAPK signaling (Figure 5B). Likewise, plant-pathogen interaction and phenylpropanoid biosynthesis were highly enriched KEGG pathways of Cluster III (Figure 5B). However, analysis of the downregulated DEGs within Cluster V showed that in general photosynthesis, carbon fixation and carbon metabolism were the main KEGG pathways (Figure 5B). Summarizing the results of the functional analysis of the host genes that show differential expression within the main *Cmm*-responsive Clusters, three biological processes deserved special attention. First, the plant-pathogen interaction molecular network was activated in the tomato-*Clavibacter* pathosystem. Second, photosynthesis together with the peripheral physiological processes was shut down. Third, the DEGs involved in phenylpropanoid biosynthesis were scattered in Clusters II and III of the upregulated genes, or in Cluster V of the downregulated genes.

### 2.5. Tomato Activated the Plant Defense Mechanisms against Cmm

**Figure 6:**
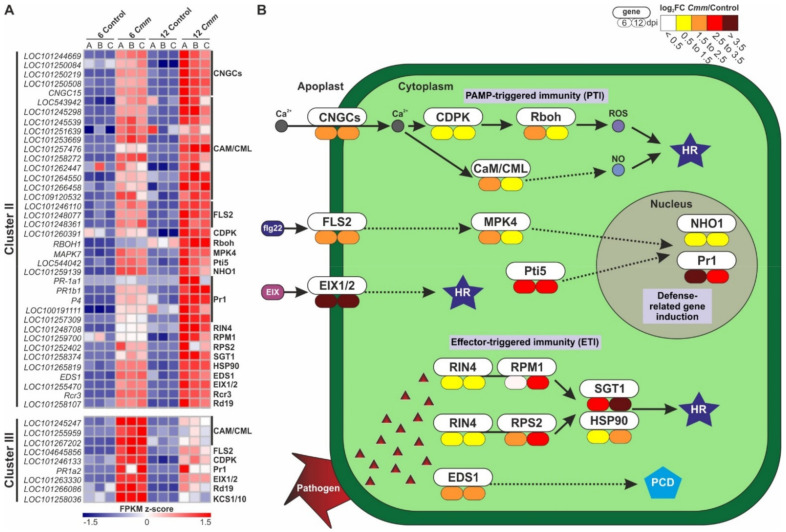
Host genes of the plant-pathogen interaction pathway activated by *Cmm*. (**A**) Heat map visualizing the stimulated expression pattern of genes involved in the plant-pathogen interaction pathway of Clusters II and III. (**B**) Graphical presentation of the expression fold change (*Cmm*/control) of genes controlling plant defense-immunity pathways at 6 and 12 dpi. HR: hypersensitive response; PCD: programmed cell death.

Based on the functional analysis of tomato responsive genes, 47 upregulated DEGs were identified being molecular components of the plant-pathogen interaction pathway. To further comprehend the response of this pathway, first, a heatmap of the level of gene expression was generated relying on the FPKM values of the transcriptome analysis (Figure 6A, Supplementary Dataset S1). Cluster II included the majority of transcripts, 38 of 47 DEGs, which showed a gradual upregulation of expression at both time points upon infection reaching a maximum level at 12 dpi. Cluster III included 9 genes that were predominantly upregulated at 6 dpi and subsequently were downregulated at 12 dpi. Based on the response pattern of these differentially expressed transcripts of *Cmm* infection, genes lying within Cluster III supported a primary mode of molecular response, whereas the genes of Cluster II modulate a secondary response.

To comprehend the way tomato plants responded to *Cmm* infection, we made an atlas of the host key responsive genes involved in plant innate immunity. The first plant line of defense depends on membrane-embedded receptor-like kinases (RLKs), receptor-like proteins (RLPs) and receptor-like cytoplasmic kinases (RLCKs) [18]. These components function as sensors of MAMPs for the perception of plant pathogens resulting in the activation of MAMP-triggered immunity (MTI). The activation of MTI is achieved via calcium signaling and MAPK cascades. Interestingly, *flagellin-sensing 2* (*FLS2*), a multi-domain transmembrane leucine-rich repeat RLK, was induced upon *Cmm* infection at both 6 and 12 dpi (Figure 6B, Supplementary Dataset S1). FLS2 recognizes a conserved N-terminal 22-amino acid sequence (flg22) of bacterial flagellin as a MAMP initiating MTI. Consistent with this, FLS2 led to induction of *mitogen-activated protein kinase 4* (*MPK4*) activating the expression of *NHO1* and *PR1*, encoding a glycerol kinase and pathogenesis-related protein 1, respectively. As MAMPs result in elevation of cytoplasmic Ca^2+^ concentration, the *cyclic nucleotide-gated channels* (*CNGCs*) were activated (Figure 6B, Supplementary Dataset S1). This led to calcium signal transmission through the induction of *calcium-dependent protein kinase* (*CDPK*) and the respiratory burst oxidase homolog protein (Rboh) to generate a burst of reactive oxygen species (ROS) [20]. In addition, the calmodulin (CaM)/calmodulin-like proteins (CML) were induced to produce nitric oxide (NO) and induced defense responses through a sequence of biochemical reactions. *Cmm* also activated the transmembrane RLPs, *EIX1* and *EIX2* (*EIX1/2*). These proteins usually recognize fungal MAMPs such as the ethylene-inducing xylanase (eix) polypeptide transmitting the signal of pathogen attack and switching on the hypersensitive response (HR) to potentially halt the pathogen before it is established within the plant host.

In general, the second line of defense against gram-negative bacteria is mediated by the host plant resistance (R) proteins that detect specific pathogen effectors in the cytoplasm and is known as the effector-triggered immunity (ETI) pathway [19,21,22,23]. Even though a typical ETI is ambiguous in the *Clavibacter*-host interaction due to the absence of the type 3 secretion system [11,24], *Cmm* infection intriguingly caused the upregulation of major genes engaged in the ETI pathway. In particular, the expression of genes encoding for the *RPM1-INteracting protein 4* (*RIN4*), two *R* genes (*RPM1* and *RPS2*), and two disease-resistance associated genes (*SGT1* and *HSP90*) were induced (Figure 6B, Supplementary Dataset S1). This response is often accompanied by a localized programmed cell death known as the hypersensitive response (HR) and is effective against host-adapted pathogens. A complementary response seemed to be activated by an enhanced disease susceptibility protein (EDS1), which was also up-regulated.

The expression pattern of DEGs identified at 6 and 12 dpi suggested a dual-mode of molecular response regarding the *Cmm*-tomato interaction. Interestingly, a primary response mechanism consisting of MTI-like genes showed a high degree of activation at 6 dpi, and remained still active at 12 dpi, albeit gene expression was significantly reduced. On the contrary, during the progression of the disease, a secondary response mechanism was highly induced at 12 dpi and coincided with the decrease in chlorophyll levels. These results are in agreement with the widely appreciated notion that plants generally activate a prime response relying on surface-localized receptor proteins, which in the case of virulent pathogens such as *Cmm* is followed by disease progression. 

### 2.6. Photosynthesis Was Gradually Reduced by Cmm Infection

**Figure 7:**
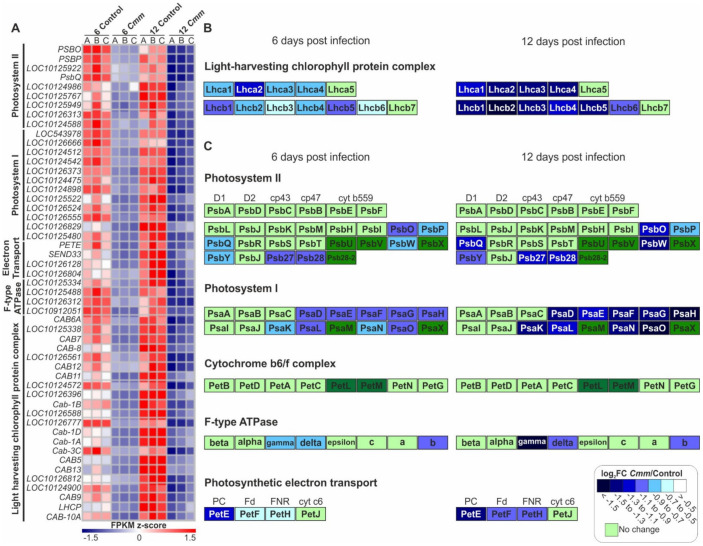
Tomato genes encoding protein components of the photosynthesis complexes were downregulated by *Cmm*. (**A**) Heat map visualizing the downregulation of genes involved in photosynthesis of Cluster V. (**B**,**C**) Comparative presentation of *Cmm* infection on the expression fold change (*Cmm*/control) of genes encoding components of tomato light harvesting complexes (**B**) and photosynthetic apparatus (**C**). In (**C**), the cells in dark green color represent components of tomato photosynthesis that has not been annotated yet.

While the host defense mechanisms were activated, the functional analysis suggested that the process of photosynthesis was deactivated by *Cmm* infection. Of a total of 5527 DEGs, 51 genes of Cluster V involved in light-harvesting chlorophyll complexes and photosynthesis were downregulated (Figure 7A, Supplementary Dataset S2). These genes were first affected at 6 dpi but a suppression of gene expression was mostly evident at 12 dpi indicating a progressive shut down effect due to the dynamic reprogramming of the host transcriptome by *Cmm*. In particular, the majority of the components of the light-harvesting chlorophyll protein complexes, LCHII and LCHI, transferring the energy of photons to the attached complexes of Photosystem II (PSII) and Photosystem I (PSI), respectively, showed a slight reduction of expression at 6 dpi (Figure 7A,B). Furthermore, the expression of the same components of LCHII and LCHI was severely affected at 12 dpi showing a reduction ranging from 50% to 75% (Figure 7B, Supplementary Dataset S2).

Likewise, a notable effect of *Cmm* infection was evident on the expression of genes encoding components of the photosynthetic apparatus (Figure 7A, Supplementary Dataset S2). PSII controls photolysis of water, releasing oxygen and generating protons and electrons. Approximately one-fourth of the PSII components, 7 out of 27, were slightly downregulated at 6 dpi and significantly affected at 12 dpi (Figure 7C, Supplementary Dataset S2). More than half of the PSI components, 9 out of 16, showed reduced expression levels at 6 dpi and enhanced downregulation at 12 dpi. At 6 dpi, notably 3 PSII components out of 27 showed about a two-fold downregulation of expression (log_2_FC < 1.0) and this number increased to 6 at 12 dpi (Figure 7C, Supplementary Dataset S2). Although, almost half of the PSI components, particularly 7 out of 16, showed reduced expression of approximately two-fold (log_2_FC < 1.0) at 6 dpi, only two additional genes were downregulated at 12 dpi. The comparative analysis of these results indicated that PSII gene expression was affected more at 12 dpi compared to 6 dpi. This transcriptional downregulation of PSII components suggests that at 6 dpi, PSII most likely modulates electron transfer towards PSI to activate a retrograde signaling that coincides with an MTI-like reaction. Conversely, downregulation of PSII components gene expression at 12 dpi potentially switched the retrograde signaling to trigger cell death [29]. Intriguingly, the expression of components of the cytochrome b6/f complex that transfer electrons from PSII to PSI and transport protons from the stroma of the chloroplast to the lumen of thylakoids, were not affected. Additionally, the structural components of the photophosphorylation system of F-type ATP synthase showed a lower level of expression compared to control plants. Taking these results into consideration, it appears reasonable that *Cmm* affects the expression of genes controlling the synthesis of NADPH and ATP, necessary for carbon fixation through the Calvin–Benson cycle.

### 2.7. Cmm Infection Caused a Two-Step Switch off Mode of Tomato Genes in Control of Phenylpropanoids Metabolism

**Figure 8:**
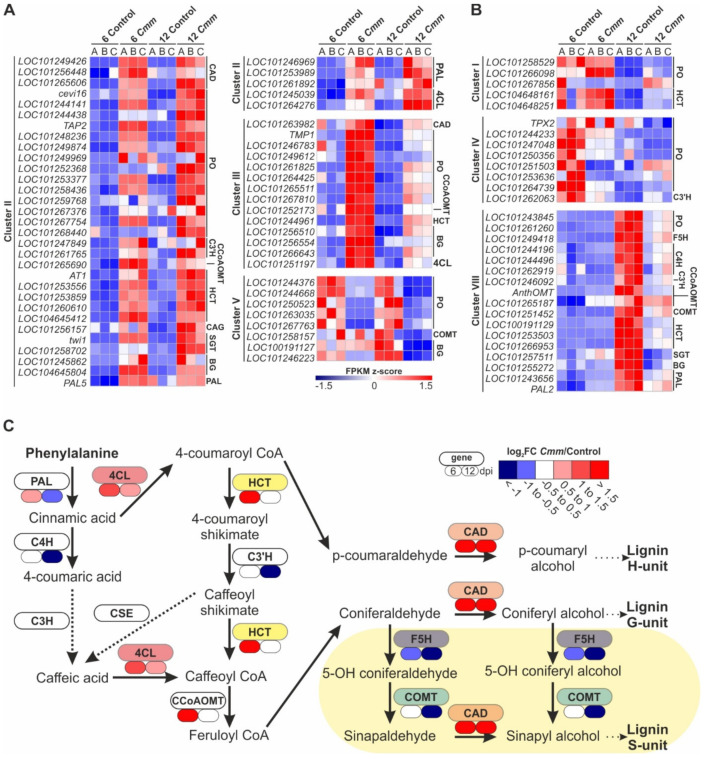
*Cmm* infection differentially regulated the tomato phenylpropanoid biosynthetic pathway genes. (**A**,**B**) Heat map of tomato DEGs involved in phenylpropanoid metabolism. (**A**) Genes within Clusters II, III and V that were constantly up- or down-regulated at both time points of *Cmm* infection. (**B**) Additional genes of the pathway without a constant expression pattern enclosed in Clusters I, IV and VIII. (**C**) Presentation of the relative expression change of genes encoding key enzymes of the monolignol biosynthesis pathway. In phenylpropanoid metabolism, the genes that were differentially expressed upon *Cmm* infection leading to hydroxycinnamyl alcohols which are polymerized into lignin are: *L-Phenylalanine Ammonia-Lyase* (*PAL*), *Cinnamic acid 4-Hydroxylase* (*C4H*), *4-hydroxycinnamate CoA Ligase* (*4CL*), *Hydroxycinnamoyl CoA:shikimate hydroxycinnamoyl Transferase* (*HCT*), *Coumaroyl shikimate 3′-Hydroxylase* (*C3′H*), *Caffeoyl CoA 3-O-MethylTransferase* (*CCoAOMT*), *Ferulic acid/coniferaldehyde 5-Hydroxylase* (*F5H*), *Caffeic acid/5-hydroxyconiferaldehyde 3/5-O-MethylTransferase* (*COMT*) and *Cinnamyl Alcohol Dehydrogenase* (*CAD*). The lower branch of the pathway demonstrating the suppressed genes at 6 dpi, modulating the synthesis of syringyl (S) lignin units, is highlighted.

Lignin is the most ubiquitous aromatic polymer that reinforces the thickening of secondary cell walls and thus acts as a physical and chemical barrier against pathogen invasion. Our functional analysis revealed tomato genes responsive to *Cmm* infection involved in the phenylpropanoid biosynthesis pathway that leads to hydroxycinnamyl alcohols formation, which can subsequently be polymerized into lignin (Figure 8A, Supplementary Dataset S3). These genes were classified into three Clusters. Clusters II and III, included 35 and 14 mainly upregulated transcripts, respectively, whereas 8 genes within Cluster V showed decreased levels of expression at both time points.

Lignin biosynthesis is a perplexed, likely nonlinear pathway beginning from L-phenylalanine mainly derived from plastids [30]. The initial steps of phenylpropanoid metabolism are controlled by specific enzymatic reactions, while several enzymes are involved in the final steps leading to lignin polymerization (Supplementary Figure S6). The end products of phenylpropanoid biosynthesis are *p*-hydroxyphenyl (**H**), guaiacyl (**G**) and syringyl (**S**) units, derived from the three primary monolignols p-coumaryl, coniferyl and sinapyl alcohols, respectively (Supplementary Figure S6). To gain a better understanding of the hubs that control the synthesis of lignin units upon *Cmm* infection, additional tomato transcripts apart from Clusters II, III and V were identified by the transcriptome analysis and introduced in the phenylpropanoid biosynthesis pathway (Figure 8B, Supplementary Dataset S3). These were 30 genes included in Clusters I, IV and VIII without a clear trend in terms of gene expression upon both time points of *Cmm* infection. 

The incorporation of transcriptomic data in the phenylpropanoid pathway revealed a differential expression pattern of genes controlling key enzymatic reactions of lignin biosynthesis and composition (Figure 8C). Notably, genes being first in the higher branches of the pathway were activated at 6 dpi, namely *L-Phenylalanine Ammonia-Lyase* (*PAL*), *4-hydroxycinnamate CoA Ligase* (*4CL*), *Hydroxycinnamoyl CoA:shikimate hydroxycinnamoyl Transferase* (*HCT*) and *Caffeoyl CoA 3-O-MethylTransferase* (*CCoAOMT*). This is an expected molecular reaction, as defense-induced lignification is a conserved basal defense mechanism in plants against pathogens in a wide range of plant species [31]. Nevertheless, at 6 dpi, the genes controlling the lower branches of syringyl (S) lignin unit synthesis were suppressed. In particular, the expression of *Ferulic acid/coniferaldehyde 5-Hydroxylase* (*F5H*) and *Caffeic acid/5-hydroxyconiferaldehyde 3/5-O-MethylTransferase* (*COMT*) genes were downregulated. On the contrary, *cinnamyl alcohol dehydrogenase* (*CAD*) controlling the downstream nodes towards the synthesis of p-hydroxyphenyl (H) and guaiacyl (G) units of lignin was highly expressed. These results supported a shift in the homeostasis of lignin units produced at 6 dpi of *Cmm* infection. While the pathway was generally induced, a clear preference to shut down the genes of S-units of lignin relatively to H- and G-units was evident.

Interestingly, an opposite response was observed at 12 dpi. While the genes encoding key enzymes of the higher branches of phenylpropanoid biosynthesis were stimulated at 6 dpi, an obvious shut down of these genes was observed at 12 dpi. Among these, it is important to emphasize the suppression of *Cinnamic acid 4-Hydroxylase* (*C4H*) and *Coumaroyl shikimate 3′-Hydroxylase* (*C3′H*), in addition to *PAL*, the first gene of the pathway (Figure 8C). Taking these points into consideration, it appears reasonable that while the *Cmm* infection is spreading, the expression of genes modulating the lignin-mediated line of defense is eventually shut down, a situation that clarifies the devastating effects of *Cmm* on plant organs and development.

## 3. Discussion

Bacterial canker is a systemic vascular disease of tomato caused by *Clavibacter michiganensis* subsp. *michiganensis* (*Cmm*). This gram-positive pathogen often generates outbreaks with devastating effects on tomato production worldwide. As climate models predict that the spread of plant diseases will be enhanced by weather instability, it is urgent to comprehend the molecular networks controlling tomato responses against *Cmm* infection. To accomplish this task, an infection system of tomato with *Cmm* was successfully developed producing reproducible results and leading to a coordinated sequence of symptom emergence. The tomato infected stems showed a typical bacterial stem canker symptom three days after *Cmm* infection, while plants’ condition was deteriorating in time leading to destruction of organ structure.

In planta bacterial growth presented a peak at the sixth day, which was maintained until day twelve, while on the fifteenth day, a gradual decrease of bacterial population was observed, most likely associated with the local necrosis of the stem. Hence, given the highest bacterial population in the plant body, 6 and 12 days of post inoculation were set as the critical time points to follow the response of the host. Analysis of the plant host transcriptome is a useful approach to understand at the molecular level the plant response to pathogens attack and ultimately to effectively control *Cmm* infection. To identify the tomato genes that respond upon *Cmm* infection, molecular analyses were performed by applying microarray [28] and cDNA-amplified fragment length polymorphism (AFLP) methodologies [32]. Recent reports highlight RNA sequencing (RNA-seq) as the most advanced and powerful tool for characterizing the transcriptome of plants upon phytopathogen infections, revealing crucial genes involved in major processes including cell wall synthesis, secondary metabolism and signaling pathways [33,34,35]. To gain a deep view of tomato progressive response against *Cmm*, a holistic transcriptome analysis was performed to enlighten the defensive molecular responses of tomato.

The RNA-seq analysis identified 5527 DEGs upon *Cmm* infection. The expression pattern of tomato genes in *Cmm* infected stems showed a higher degree of correlation at both time points, supporting the high impact of *Cmm* infection in reprogramming the tomato transcriptome. Furthermore, the majority of tomato genes were upregulated, reflecting the activation of major plant responses against pathogen attack. Among the most highly induced tomato genes at 6 dpi were these encoding pathogenesis-related proteins and transcription factors namely *SBTP69B*, *SBT1.7*, *PIP1, HSR4* and *WRKY40*. The *WRKY* family of transcription factors is known to participate in JA, SA, and ABA signaling to regulate defense responses [36], whereas *PIP1* gene responds to potential virulence factors [37]. Interestingly, a subset of identified genes associated with ethylene synthesis and response, including *ACO1*, *ACO5*, *Pr4* and *Pr6*, were also reported in a previous tomato microarray analysis upon *Cmm* infection [28]. A common group of highly expressed host genes was identified by both RNA-seq and microarray analyses. Nevertheless, our RNA-seq analysis provides deep understanding of the tomato-*Clavibacter* pathosystem (~45-fold higher number of DEGs), which downregulates host’s photosynthesis and triggers distinct timing defense response processes revealing among others an interplay with defense lignin metabolism.

Apart from oxygenic photosynthesis, chloroplasts integrate environmental signals and act as metabolic hubs. Moreover, chloroplasts contribute to plant immunity through retrograde signaling pathways by perceiving and responding to biotic stresses [29]. Functional analysis of tomato DEGs upon *Cmm* infection identified Clusters of genes involved in photosynthesis showing downregulation of expression at both time points, albeit at 12 dpi the effect was more dramatic. Notably, the expression of genes encoding components of both light-harvesting chlorophyll protein complexes, LCHI and LCHII, showed the highest reduction. Among the two photosystems, at 6 dpi most components of PSI were downregulated compared to PSII, which remained relatively stable. On the contrary, PSII components compared to PSI showed a significant downregulation of expression at 12 dpi. Based on recent findings, a possible explanation of this shift is that at 6 dpi PSII likely tuned a retrograde signaling that coincides with an MTI-like response, while at 12 dpi the retrograde signaling was associated with cell death [29]. Components of electron transport and the photophosphorylation system showed a high degree of reduction, pinpointing a negative effect of *Cmm* on the terminal products of photosynthesis light reactions, namely NADPH as reducing power and ATP. The results of the gene expression analysis were further supported by the reduced level of chlorophylls, the main photosynthetic pigments, confirming that photosynthesis is often significantly affected in many plant systems in response to biotic conditions [38,39]. Hence, collecting data related to the level of chlorophylls, could provide important information about the macroscopic health status of tomato plants in the open field or the greenhouse. This smart farming approach will provide valuable information initiating precision agriculture operations and management to constrain *Cmm* spread. Concomitantly, the energy loss accompanied by photosynthesis reduction upon pathogen attack has been reported to trigger the activation of defense-related components until the pathogenic growth is suspended [40]. Consequently, the expression of defense-related genes and the production of plant secondary metabolites associated with plant resistance against pathogens often become the plant’s top priorities [29]. Interestingly, our study identified major reprogramming of tomato gene expression towards the coordination of antimicrobial defense mechanisms and the phenylpropanoid biosynthesis, providing evidence supporting a tight interplay between these two defense-induced responses.

Initially, MTI activation takes place in the apoplast depending on calcium signaling and MAPK cascades [18,20,21,22,23]. As a secondary messenger, Ca^2+^ after pathogen infection activates the oxidative burst and PR gene expression [20,41,42]. At both 6 and 12 dpi upon *Cmm* infection, a response mechanism was activated reminiscent of the MTI pathway. Tomato genes encoding both the *FLS2* receptor-like kinase, a major plant PRR that recognizes one of the most common MAMPs, the bacterial flagellin (flg22), and *CNGCs* cyclic nucleotide-gated ion channels involved in pathogen-inducible Ca^2+^ influx, were upregulated. Nevertheless, the *Mitogen-activated Protein Kinase 4* (*MPK4*), was mainly upregulated at 6 dpi, leading to induction of defense-related genes *Pathogenesis related 1* (*Pr1*) and *NonHOst1* (*NHO1*) expression. Interestingly, *Arabidopsis* has shown increased pathogen resistance against bacterial pathogen infection when *MPK4* is overexpressed [43]. Similar to MPK4, the signaling pathway of Ca^2+^, acting downstream of CNGCs, was higher expressed at 6 dpi compared to 12 dpi as calcium signaling components including the *Calcium-Dependent Protein Kinase* (*CDPK*) and *CalModulin* (*CaM*)/*CalModulin*-Like proteins (*CML*), supporting that a primary response mechanism was initially activated upon *Cmm* infection. These signaling components eventually lead to the production of Reactive Oxygen Species (ROS) and Nitric Oxide (NO) inducing the defense response [44].

A second line of defense of the plants is the effector-triggered immunity (ETI) [19,20,21,22,23], which is triggered when plant cytoplasmic resistance (R) proteins recognize these “effector” proteins. While *Cmm* lacks a type 3 secretion system (T3SS) to activate a typical ETI pathway in the *Clavibacter*-tomato interaction [11,24], genes engaged in cell death initiation were also differentially expressed at both time points, albeit most of them were predominately upregulated at 12 dpi. Particularly, the *RPM1-INteracting protein 4* (*RIN4*), two *R* genes, *RPM1* and *RPS2*, and two disease-resistance associated genes, the *Suppressor of the G2 allele of skp1* (*SGT1*) and *Heat-Shock Protein 90* (*HSP90*) were higher expressed at 12 dpi compared to 6 dpi. Mutations of these genes totally disabled plant defense conferred by Resistance (*R*) genes [45], which in line with our results, signifies an important role of basal plant defense responses against the gram-positive bacterium *Cmm* in tomato. The constant activation of *EIX1/2* plant receptors, together with the upregulation of *Enhanced Disease Susceptibility* (*EDS1*) gene, led to a localized programmed cell death. This response was supported by the results of trypan blue staining of stems indicating the cumulative triggering of cell death reaching its highest point at 12 dpi to block, through plant cell death, the pathogen growth and spread. Taken together, our experimental system reveals a subtle chronological difference regarding the activation of antimicrobial defense mechanisms. Furthermore, even though a typical ETI has not yet has been determined, *Cmm*-infection triggers the expression of genes engaged in cell death formation, revealing a prevailing route towards plant defense. 

Analysis of transcriptomic data also unraveled the role of lignin to strengthen further tomato disease resistance. Lignin is not only essential for structural integrity but also protective, acting as a mechanical barrier to pathogen attack and as a chemical barrier for cell wall degrading enzymes of microbial origin [46,47]. Hence, lignification is among the basal responses of a plant host to counteract a pathogen infection [30,31,48]. In angiosperms, lignin is typically composed of Guaiacyl (G) and Syringyl (S) units, whereas low traces, less than 5%, occur as *p*-Hydroxyphenyl (H) units [30,49]. Upon *Cmm* infection, the pathway leading to monolignol biosynthesis, was highly activated at 6 dpi as was shown by the induction of a deaminase, L-phenylalanine ammonia-lyase (*PAL*). PAL is the first enzyme of the pathway to convert L-phenylalanine to cinnamic acid, followed by a series of hydroxylases, O-methyltransferases and reductases. Our findings confirmed the induction of the pathway supporting the critical role of PAL in lignin synthesis and defense. Consistently, overexpression of *PAL* enhanced plant resistance against pathogens [50], whereas loss of *PAL* gene function or downregulation of expression by RNAi-mediated gene silencing led to decreased penetration resistance to pathogens and increased plants susceptibility [51,52,53]. In addition to *PAL*, three other central genes were upregulated at 6 dpi, namely *4CL*, *HCT* and *CCoAOMT* supporting that the monolignol pathway was turned on by *Cmm* infection. This conclusion is in agreement with previous genetic and physiological studies indicating that gene silencing of *HCT* caused severe loss of lignified structure integrity [54,55] and that of *CCoAOMT* compromised penetration resistance against pathogens [51]. Likewise, upregulation of *HCT* in infected maize plants showed increased lignin accumulation [56].

While in general the expression of key genes within the higher branches of the phenylpropanoid pathway was stimulated by *Cmm* infection at 6 dpi, the genes leading to the synthesis of S-lignin were suppressed. In particular, both *F5H* and *COMT* genes were downregulated most likely shifting the homeostasis between the three terminal lignin units supporting the notion that S-lignin contributes to less extent to plant defense. Furthermore, considering the minor amounts of H-lignin, the defense-induced lignin should be enriched in G-lignin, and thus the molar ratio of S- to G-units (S/G) would determine the composition of defense lignin [30,49]. Genetic evidence supported this molecular reprogramming of tomato genes to resist *Cmm* infection. The *fah1-2* mutant, deficient in F5H activity, which is involved in the branch of the monolignol pathway generating the S-units, accumulated wild-type levels of total lignin but displayed a loss of specifically S-lignin, as the flux to S-lignin was redirected to G-lignin [57,58,59,60]. On the contrary, upregulation of F5H activity through gene overexpression diverted the flux away from G-lignin biosynthesis towards S-lignin [58]. The growth phenotypes of *fah1-2* mutant and transgenic plants were similar to these of wild-type plants, indicating that the changes in lignin composition were exclusively associated with defense responses [61]. Similar to the *fah1-2* mutant, the S/G ratio was strongly reduced in a mutant with *COMT* loss of function, as the result of a large reduction in S-units [62]. The near total loss of S-units in *comt* mutant was also not associated with growth defects [55]. 

Interestingly, in a recent analysis on lignin biosynthesis, the *pal1-3* and *cse-2* mutant alleles within the higher branches of lignin biosynthesis displayed reduced overall lignin content and thus were susceptible to bacterial infection [48]. In contrast, completely devoid of S-lignin *fah1-2* and *omt1-2* mutants deficient in F5H and COMT activity, respectively, albeit showed normal lignin levels, they did not become susceptible to bacterial infection [48,63]. The wild-type level of both *fah1-2* and *omt1-2* mutant resistance to pathogen infection supports the notion that G-lignin is the major lignin unit contributing to basal plant immunity. Our findings provide insights into the transcriptional control of monolignols pathway upon pathogen invasion supporting that synthesis is diverted away from S-lignin. In conjunction with our data, the S-rich lignin is less condensed compared to G-rich lignin, which is more cross-linked [64,65]. Hence, the defense-induced lignin, predominantly comprised of G-units, seems to be less prone to degradation by microbial enzymes than S-rich lignin and thus building a better defensive barrier against pathogens.

The increased G-lignin content in *Arabidopsis*, through *COMT* dependent downregulation of the monolignol pathway, has led to reduction of RPS-mediated resistance to bacterial pathogens, indicating inactivation of ETI defense [48,66,67]. Consistently, induction of genes engaged in G-lignin monomers at a cost of S-lignin biosynthesis was detected in *Cmm*-infected tomato plants. Interestingly, our results support a tight interplay between the monolignol biosynthetic pathway and the distinct activation of basal immune responses of tomato upon *Cmm* infection. A primary defense response was synchronized with a change of lignin synthesis towards G-units enrichment to build up an early defensive barrier against *Cmm* at 6 dpi. This positive interplay is congruent with the role of *MYB15* transcription factor, which is sufficient for *flg22*-induced lignification by activating genes involved in the biosynthesis of G-lignin, but not S-lignin [48]. In striking contrast, a near-total shut down of the phenylpropanoid pathway, which was observed late at 12 dpi, beginning with *PAL* downregulation, led to the subsequent activation of a secondary defense mechanism. The rationale of this outcome is the combinatorial interaction of defense lignin enriched in G-units and of a primary defense layer, at 6 dpi with *Cmm*, whereas the suppression of total lignin production at 12 dpi coincides with the activation of a secondary defense layer. Hence, as monolignol biosynthesis collapsed, a secondary defense mechanism became active potentially being catastrophic for both the host infected cells and the bacterial invader. 

In conclusion, the transcriptome profiling of tomato performed in this study revealed the gene networks that respond against *Cmm* infection to facilitate the design of effective disease management strategies. We show that the transcriptional downregulation of PSI and PSII components most likely represent a timing issue as a molecular switch between the primary and secondary defense response coincides with the activation of an MTI-like and cell death pathway, respectively. Furthermore, alterations of gene expression involved in lignin biosynthesis are associated with the activation of distinct tomato immune defense responses against *Cmm*. Taking into account the rapid and strong resistance response of such specific defense mechanisms, our findings open a new perspective concerning *Cmm* pathogenicity control by generating pathogen-tolerant tomato cultivars with enhanced defense through fast-track breeding. Future research is required to examine whether the change in monolignols balance is associated with tomato resistance against *Cmm* infection.

## 4. Materials and Methods

### 4.1. Plant Material, Growth Conditions and Bacterial Strains 

Tomato (*Solanum lycopersicum*) Ekstasis F1 hybrid seeds (Nirit seeds Ltd., Moshav Hadar Am, Israel) were planted in plastic pots containing Potgrond P, a natural turf mixture (Klasmann-Deilmann GmbH, Geeste, Germany), and grown at 22 °C in a Fitotron (Weiss Gallenkamp, Loughborough, UK) growth chamber under a long day photoperiod with 16 h of light and 8 h of darkness per day and 100 μmol m^−2^ s^−1^ light intensity. For this study, an indigenous bacterial strain of *Clavibacter michiganensis* subsp. *michiganensis* (*Cmm*), isolated from Pomodoro tomato cultivar in Tripoli (37.5101° N, 22.3726° E), Greece, was used. Identification of the *Cmm* strain was based on Gram-positive stain test, PCR molecular characterization with specific primers [68,69], immunostaining with *anti-Cmm* polyclonal antibody (LOEWE Biochemica GmbH, Sauerlach, Germany) using FITC-conjugated secondary antibody (Biotium Inc., Fremont, CA, USA) and tomato pathogenicity tests.

### 4.2. Inoculation Procedure and In Planta Bacterial Growth Analysis

*Cmm* bacteria were grown for 36 h at 26 °C in a rotating shaker, in YPDNM medium containing 1% Yeast extract, 2% Peptone, 2% D-Glucose, 1% NaCl and 10 mM MgCl_2_. Bacteria were pelleted by centrifugation at 1200× *g* for 10 min, washed, and diluted to a titer of 10^8^ cfu/mL in dilution buffer (10 mM MgCl_2_, 10 mM Tris-HCl pH:6.8). Bacterial suspensions or dilution buffer were injected into the stem region between the cotyledons of 2-week-old plants with a syringe fitted with a 30-gauge needle. In planta bacterial growth was measured for 18 days at 3-day intervals by grinding three stem pieces (1 cm in length) in Dilution buffer and plating serially diluted samples on YPDNM medium plates. Each stem piece was derived from an independent plant and was cut at 1 cm above the inoculation site. After incubation of the plates at 28 °C for 5 days, the number of colony-forming units per gram of fresh tissue (cfu/g) was determined for each sample.

### 4.3. RNA Extraction, RNA Sequencing and Transcriptome Analysis 

RNA preparation and RNA-seq analysis were performed as previously described [70]. Briefly, total RNA was extracted using the Direct-zol RNA Miniprep kit (Zymo research, Irvine, CA, USA) with an on-column DNase treatment according to the manufacturer’s instructions from control and *Cmm* infected tomato stem sections of 1 cm in length, 1 cm over the injection site, at 6 and 12 dpi. Stems of 5 plants were pooled for RNA isolation of each sample. The samples were analyzed in triplicates. The quantity and quality of RNA were assessed using a NanoDrop 1000 spectrophotometer and agarose gel electrophoresis. RNA-seq libraries were generated using the TruSeq Low Input kit according to the manufacturer’s instructions (Illumina). Sequencing was performed on BGISEQ-500 platform instrument at BGI (Beijing Genomics Institute, Shenzhen, China) of the three biological replicates of each sample. Raw reads were filtered into clean reads and aligned to the Tomato genome (GCF_000188115.3_SL2.50_ncbi_20180905). RNA-seq data were analyzed using the SOAPnuke (version v.1.5.2) with parameters ‘‘-l 15, -q 0.4, -n 0.1″ and the HISAT2 pipeline (version 2.0.4) with parameters ‘‘--phred33, --sensitive, --no-discordant, --no-mixed. -I 1, -X 1000″. Approximately 138 million reads were obtained for the control or *Cmm* infected samples at both time points (6 and 12 dpi). On average, about 94.71% of the clean reads could be unambiguously aligned to the SL2.50 reference genome sequence. Clean reads were mapped to reference Bowtie, and then the gene expression level was calculated with RSEM with default parameters. Statistical analysis of differential gene expression was conducted utilizing DESeq2. A multiple-test corrected *p*-value of 0.05 was adjusted using the Benjamini and Hochberg’s approach, resulting in adjusted *p*-value (*Padj*). Transcripts with fold change greater than 2 and a *Padj* value < 0.05, identified by DESeq 2, were assigned as differentially expressed. Hierarchical clustering and heatmap of the differentially expressed genes (DEGs) were performed with Perseus software (version 1.6.8.0) using Euclidean distancing with average linkage and without any constraints in the algorithm. To plot the heatmaps, the *z*-scores of the FPKM values were calculated in terms of standard deviations from the mean, describing a value’s relationship to the mean of each sample value.

### 4.4. Functional Annotation

Gene ontology (GO) enrichment for the differentially expressed genes (DEGs) within the clusters of interest was performed using the PlantRegMap database (http://plantregmap.gao-lab.org/go.php; accessed on 1 September 2020). GO categories were classified in terms of the biological process with *p* ≤ 0.05. For KEGG enrichment analysis, the data of transcriptome analysis of gene clusters with distinct expression pattern, were integrated on the Pathview R package to generate the gene network maps [71]. The molecular IDs of DEGs were introduced online in the form of the ENTREZ gene annotation. First, the ratio of absolute expression values of DEGs generated by DESeq2 between the *Cmm* infected and control plants was calculated for both time points and then the outcome was transformed to log_2_ fold change (FC) representing the relative expression level of each single-gene node. These values of relative gene expression were uploaded to Pathview online generating graphs in the native KEGG view style. For nodes corresponding to multiple genes, the absolute expression values of these genes were summarized to calculate the relative expression level of a multi-gene node as log_2_FC. 

### 4.5. Real-Time qPCR Analysis

Reverse transcription (RT) was performed on 3 μg of total DNA-free RNA using SMART-MMLV Reverse Transcriptase (Clontech laboratories, Mountain View, CA, USA). Quantitative gene expression analysis was performed using specific primers (Supplementary Table S4) with a PikoReal 96 Real-Time PCR System (Thermo Scientific, Waltham, MA, USA) using the SYBR Select Master Mix (Applied Biosystems, Waltham, MA, USA). For qRT-PCR analysis, *Glyceraldehyde-3-phosphate dehydrogenase* (*GAPDH*) was chosen as housekeeping gene and the control plants were used as internal calibrators. Quantification of gene expression was calculated as the expression of the gene of interest relative to *GAPDH* expression based on 2^−Δ*Ct*^ method. The fold change was expressed as the ratio of the quantified gene expression between the *Cmm* infected and control plants and calculated by the 2^−ΔΔ*Ct*^ method as previously described [72].

### 4.6. Histochemical Staining Techniques

Trypan blue staining was performed to visualize cell death. Briefly, stem sections were performed manually and stained in Trypan blue staining solution (0.1% *w*/*v*) for 5 min. The sections were rinsed and examined under a microscope. An Olympus BX-50 optical microscope mounted with an Olympus DP71 camera was used to examine and photograph the stem sections. The Cell^A software (Olympus Soft Imaging Solutions) was used for image capturing.

### 4.7. Extraction and Quantification of Photosynthetic Pigments

Photosynthetic pigments were extracted from control and Cmm infected plants for a time period of 18 days with 3-day intervals from true plant leaves. The leaves were incubated with DMSO for 30 min at 65 °C. The OD at 663 nm and 645 nm of each extract was measured and pigments concentrations (mg/gr fresh weight) were calculated as previously reported [70]. Three independent experiments were performed for each sample.

## Data Availability

The transcriptomic data from this article has been deposited in the Gene Expression Omnibus database at the National Center for Biotechnology Information (NCBI) under the accession number GSE160971.

## Supplementary Materials

The following are available online at https://www.mdpi.com/article/10.3390/ijms22168442/s1.

## Abbreviations

*Cmm*	*Clavibacter michiganensis* ssp. *michiganensis*
RNA-seq	RNA-Sequencing
FPKM	Fragments per Kilobase Million
DEGs	Differentially Expressed Genes
GO	Gene Ontology
KEGG	Kyoto Encyclopedia of Genes and Genomes
JA	jasmonic acid
SA	salicylic acid
ABA	abscisic acid
MAMP	Microbe-associated molecular patterns
MTI	MAMP-Triggered Immunity
ETI	Effector-Triggered Immunity
