# Supplementary material for: Clavibacter michiganensis Downregulates Photosynthesis and Modifies Monolignols Metabolism Revealing a Crosstalk with Tomato Immune Responses
Source: Int J Mol Sci. 2021 Aug 5;22(16):8442. doi: 10.3390/ijms22168442 (PMC8395114; doi:10.3390/ijms22168442)
Supplement: Supplementary file 1 [file ijms-22-08442-s001.zip › Supplementary Materials (Tables S1-S4 & Fig S1-S6)_v02.pdf]

International Journal of
Molecular Sciences

MDPI

Supplementary Materials

Clavibacter michiganensis downregulates photosynthesis and modifies monolignols metabolism revealing a crosstalk with tomato immune responses

Dikran Tsitsekian[1#], Gerasimos Daras[1#], Konstantina Karamanou[1], Dimitris Templalexis[1], Konstantinos Koudounas[1,2], Dimitris Malliarakis[3], Theologos Koufakis[4], Dimitris Chatzopoulos[5], Dimitris Goumas[3], Vardis Ntoukakis[6], Polydefkis Hatzopoulos[1*] and Stamatis Rigas[1*]

[1]Laboratory of Molecular Biology, Department of Biotechnology, Agricultural University of Athens, Iera Odos 75, 11855, Athens, Greece.

[2]EA2106 Biomolécules et Biotechnologies Végétales, Université de Tours, Tours, France.

[3]Laboratory of Plant Pathology-Bacteriology, Department of Agriculture, School of Agricultural Sciences, Hellenic Mediterranean University, Estavromenos, 71004, Heraklio, Greece.

[4]AGRIS S.A., Imathia Horticulture Center, Kleidi, Imathia, Greece.

[5]Biomedical Research Foundation of the Academy of Athens, Athens, Greece.

[6]School of Life Sciences and Warwick Integrative Synthetic Biology Centre, University of Warwick, Coventry, UK.

This PDF file includes: Supplementary Tables S1 to S4

Supplementary Figures S1 to S6

Supplementary Table S1. Presentation of the total clean reads per biological replicate (A-C) and the percentage of genome mapping from mock and *Cmm* infected plants.

Sample	Total Clean Reads	Total Mapping Ratio	Uniquely Mapping Ratio
**6 control A**	134,334,940	95.38%	80.54%
**6 control B**	134,348,718	95.08%	79.98%
**6 control C**	134,275,592	95.55%	80.46%
**6 *Cmm* A**	134,769,912	94.02%	73.20%
**6 *Cmm* B**	133,529,134	94.16%	73.51%
**6 *Cmm* C**	133,359,256	94.23%	73.70%
**12 control A**	135,721,462	94.27%	75.17%
**12 control B**	136,311,834	94.95%	75.72%
**12 control C**	134,152,670	95.02%	75.45%
**12 *Cmm* A**	133,249,962	94.56%	75.66%
**12 *Cmm* B**	134,586,018	94.97%	80.60%
**12 *Cmm* C**	135,089,864	94.31%	75.81%

Supplementary Table S2. Presentation of the total number of genes identified and the statistics of transcripts per biological replicate (A-C) from mock and *Cmm* infected plants.

Sample	Total Gene number	Total Transcript number
**6 control A**	23,269	33,028
**6 control B**	23,233	33,074
**6 control C**	23,225	33,055
**6 *Cmm* A**	23,631	33,662
**6 *Cmm* B**	23,678	33,770
**6 *Cmm* C**	23,675	33,791
**12 control A**	23,265	33,098
**12 control B**	23,243	32,983
**12 control C**	23,175	32,849
**12 *Cmm* A**	23,640	33,425
**12 *Cmm* B**	23,722	33,584
**12 *Cmm* C**	23,592	33,405

Supplementary Table S3. RNA-seq data of a subset of highly induced tomato genes at 6 and 12 dpi upon *Cmm* infection.

Spot	Gene ID	Symbol ID	Description	6 days		12 days	
				log_2FC (*Cmm*/control)	$-log_{10}$ Padj	log_2FC (*Cmm*/control)	$-log_{10}$ Padj
1	101245099	LOC101245099	ncRNA	8.37	263	4.41	14.62
2	778273	PIP1	*Solanum lycopersicum* phytophthora-inhibited protease 1 (PIP1)	6.87	298	4.53	16.43
3	544296	SBTP69B	*Solanum lycopersicum* subtilisin-like protease (P69B)	8.21	267	3.46	4.80
4	101261799	LOC101261799	NIM1-INTERACTING 2 [Solanum tuberosum]	8.31	275	6.52	90.60
5	101250055	LOC101250055	auxin-induced protein PCNT115-like [*Solanum pennellii*]	8.64	158	9.01	174.47
6	101268780	WRKY40	WRKY transcription factor 40	8.76	256	4.75	12.94
7	101243805	WRKY40	WRKY transcription factor 40	8.83	179	6.88	118.32
8	101250708	LOC101250708	heavy metal-associated isoprenylated plant protein 2-like	6.55	288	7.29	169.25
9	778308	SBT1.7	subtilisin-like protease SBT1.7	10.53	299	2.99	2.76
10	101245699	DC9.1	glycine-rich protein DC9.1-like	8.68	195	5.24	8.80
11	101250191	HSR4	Hyper-Sensitivity-Related 4-like	9.81	243	6.11	15.43
12	544052	ACO1	1-aminocyclopropane-1-carboxylic acid (ACC) oxidase 1	1.92	10	3.46	9.76
13	544185	Pr4	pathogenesis-related leaf protein 4 precursor	7.48	55	1.78	0.08
14	544123	Pr6	pathogenesis-related leaf protein 6 precursor	7.77	-	3.72	7.96
ACO5	543800	ACO5	1-aminocyclopropane-1-carboxylic acid (ACC) oxidase 5	-1.10	2	-1.34	1.05

Supplementary Table S4. Oligos sequences used in qRT-PCR gene expression analysis

Gene ID (RNA-seq)	Gene symbol		Primer sequence (5'-'3)	Tm (ºC)	cDNA (bp)	gDNA (bp)
		For	GGTTATTCTACTAATAATATCCTCACAAATTG	60.3		
101245099	LOC101245099	Rev	CCTCATTAGTGGTATTTCCATTCATTG	60.3	160	160
		RT	CGGATTTCTGATGTTAGCTTTAGACAC	60.3		
		For	GAGAGGGAGTGTCACAGGAGTCAAG	62.1		
778273	PIP1	Rev	ACAGTCATTAGTCCGCCCTCACAAC	58.9	155	570
		RT	TAGCCGCAATACCAACAGAAATAGG	60.8		
		For	CCTCAATGTCTTGCCCTCACCTTAG	63.5		
544296	SBTP69B	Rev	GAGCCTTTCATCGAGTATTGGACTATTAG	63.5	185	185
		RT	CTAGTCCTGGATCATTTGCCCTTGA	65.1		
		For	ATGCTACTTATGGACGGAGAAAAGA	61.8		
101261799	LOC101261799	Rev	GAACTCGTTAACCTCCGCCTCTGA	58.4	205	392
		RT	CCGTTCTCTCTTCTGTCCAGTTG	58.4		
		For	GTACAATTAGAATGGTCACTATGGT	60.5		
101250055	LOC101250055	Rev	TTATGCTCAAGATTCTCGCCCTGAA	58.4	215	405
		RT	TTGCCAATTCACAAATCCTCTCGT	60.1		
		For	CATACATCAATTTTGAAGAAGCATCAC	58.8		
10126780	WRKY40	Rev	CTTGGAGAAGGATTATCTCTTGTCAC	61.6	165	350
		RT	GGATCTGTTGTATTCACTAATGGCAC	61.6		
		For	GAGGCCAAGAGAAATCACAACCAATG	63.2		
101243805	WRKY40	Rev	GACCATATTTTCTCCAGTTATATCCATCC	61.1	110	370
		RT	GTGTTCCCCTTCATATACACCTACTA	63.4		
		For	AAGGTAGTTATTGATGTGTCTGTGA	58.4		
101250708	LOC101250708	Rev	TAACCAGCTCCACTGCATCTATTCCCT	61.8	230	560
		RT	ACTGGCTCTGGACCTTGGTTCACTA	65		
		For	AAATGATCCAGGACTAGTTTATGATACC	64.4		
778308	SBT1.7	Rev	GGGATACTTTCAACCTCCGAGCAA	64.4	145	145
		RT	ATCTGCACCGTGTAAGATGATTTGG	62.2		
		For	CTTGGCCTTTTTGTGGCTATTTTTGC	61.6		
101245699	DC9.1	Rev	CTGCTACGTCCGTCAGTATGTTC	62.4	115	250
		RT	GGTTTATATCCATTGTTGTATTCGTCAT	59.2		
		For	GCAACAAGTTATCACCTAACATTCATC	60.3		
101250191	HSR4	Rev	TAGATTGTGTTTGTCTACAGAGCCA	59.7	155	785
		RT	GTACATTCTCTCACAATCCACAGT	59.3		
		For	AGATGCTTGTGAGAATTGGGGCTTC	63.46		
544052	ACO1	Rev	CCACCTAGTTCCTTAAACCTCTGTTCCA	63.87	135	410
		RT	GATACACTTGTGTACTTTCCTCTG	59.73		
543800	ACO5	For	GGAAACATCAGCAACACAGACTG	61	170	330

		Rev	GCCAAGGTTCTCACACATTAGTTC	61.45		
		RT	ATCCTTCTTCCTCAATGCCCAT	59		
		For	CATCTCATTGTTACTCACTTGTCTC	60.1		
544185	Pr4	Rev	CTCTTGAGTTGGCATAGTTTTGTG	59.73	180	180
		RT	TAAGGACGTTCTCCAACCCAGT	61.11		
		For	CTCACTTGTCTCATGGTATTAGC	59.3		
544123	Pr6	Rev	CTTGAGTTGGCATAGTTTTGTG	56.9	165	165
		RT	CCACCCATTGTTGCAACGAGCC	64.57		
		For	CAACATCATCCCTAGCAGCACT	60.75		
101258368	GAPDH (GAPC2)	Rev	ACCCTTCAATTTACCCTCTGA	60.47	200	290
		RT	CCATATGGCAGATCAAATCAATCAC	60.01		

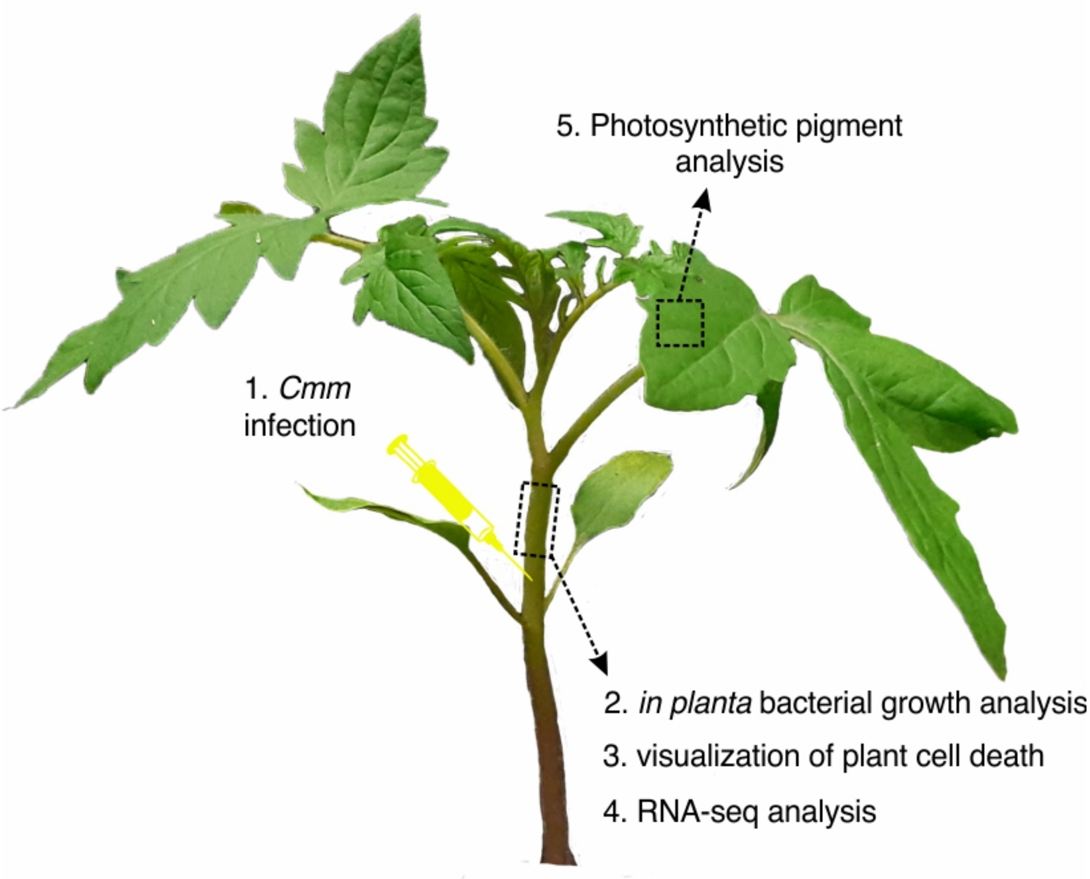

Supplementary Figure S1. Experimental workflow of tomato seedlings infected by *Cmm*. Two-week-old seedlings of Ekstasis F1 tomato hybrid variety were inoculated with a suspension of *Cmm* bacteria at a titer of 10^8 cfu/mL in the stem region between the cotyledons. The *in planta* bacterial growth analysis, transcriptome analysis and histochemical stains were performed in stem sections obtained 1cm over the inoculation site. Photosynthetic pigment analysis was performed from the true leaves of plants.

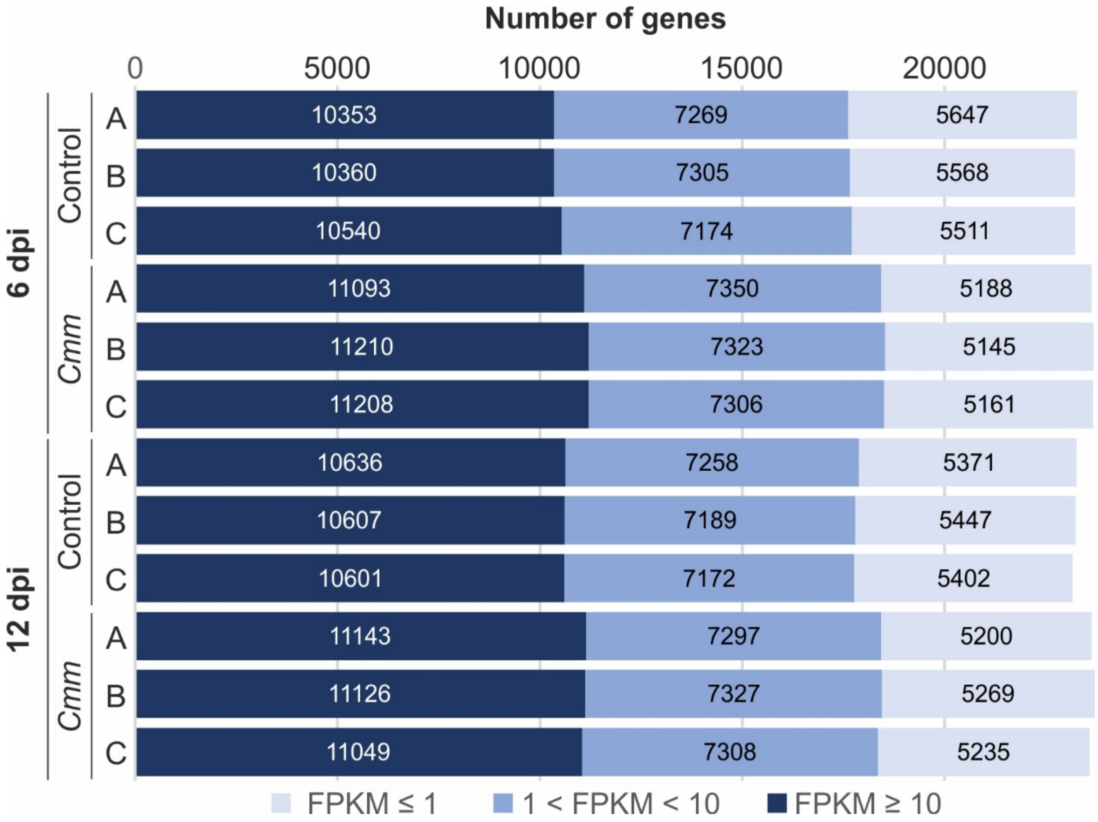

Supplementary Figure S2. Clustering of genes based on the expression level. A-C represent the three biological replicates originating from control and *Cmm* infected plants of each time point (6 or 12 dpi). X-axis represents the number of genes included in each cluster. The column with dark blue includes the highly expressed genes with FPKM ≥ 10, contrary to the column in light blue that represents the genes with low expression levels (FPKM ≤ 1). The genes with intermediate expression (1 < FPKM < 10) are shown by the medium blue column.

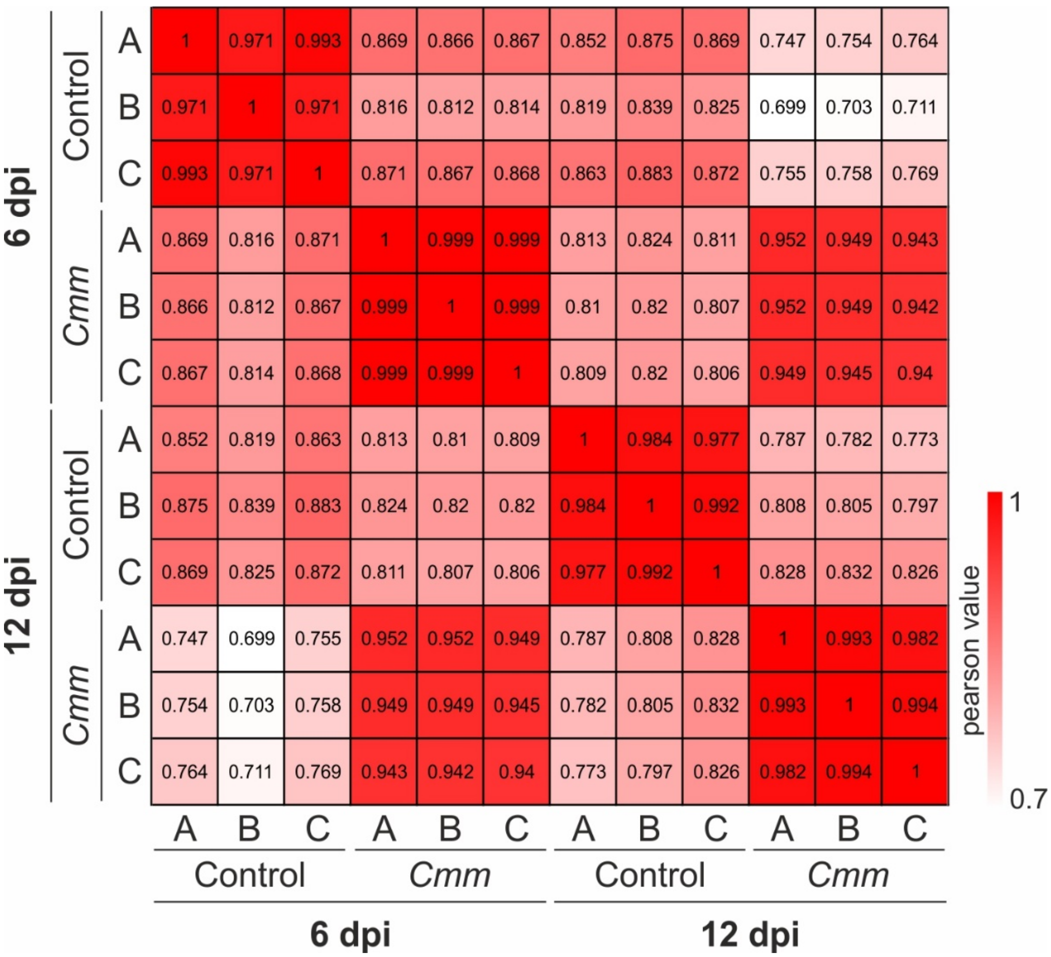

Supplementary Figure S3. Pearson correlation coefficients between the samples. The expression level of the entire gene set for each pair of samples was used to calculate the Pearson correlation coefficients, the samples were hierarchically clustered and the correlation coefficients between each sample were visually displayed as a heat map.

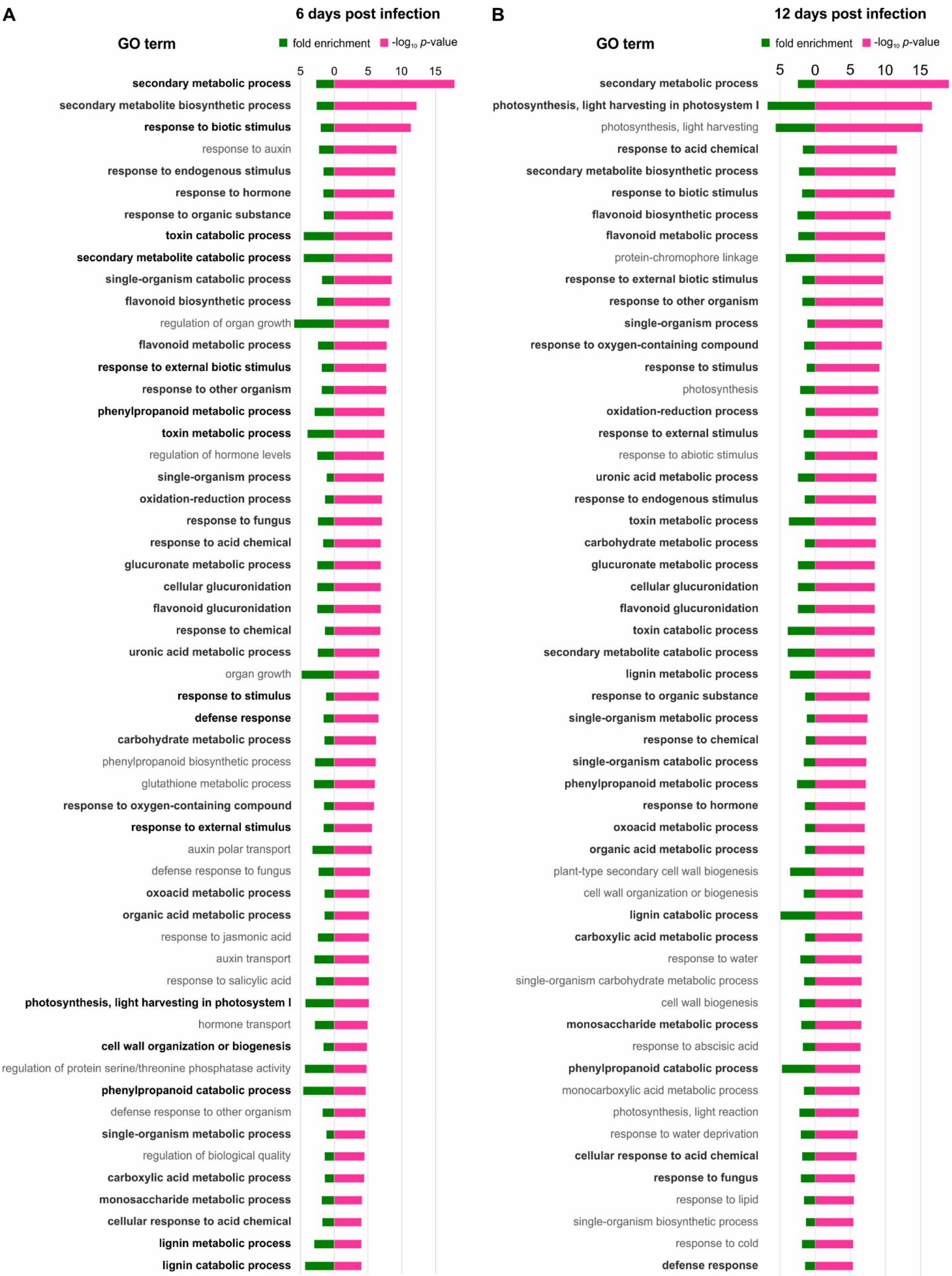

Supplementary Figure S4. Over-representation analysis of differentially expressed genes (DEGs) of both time points upon *Cmm* infection. Gene ontology (GO) analysis of DEGs at 6 (A) and 12 (B) days post infection. The terms in bold are common between the two time points.

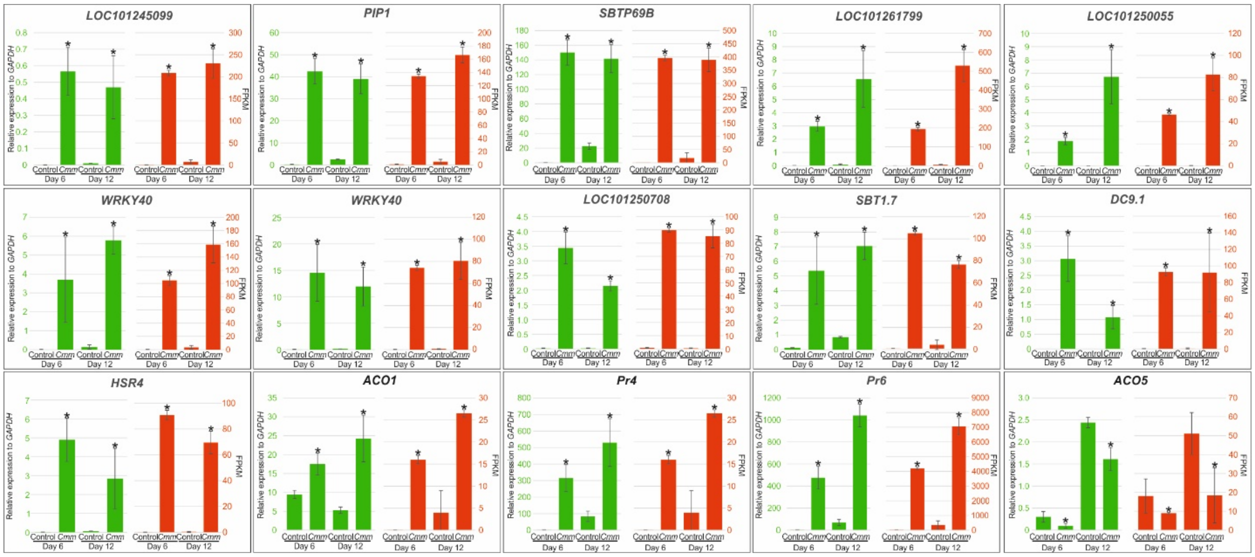

Supplementary Figure S5. Quantitative real-time PCR (qRT-PCR) validation of a subset of highly induced tomato genes upon *Cmm* infection. Columns in green show the quantified expression of each gene obtained by qRT-PCR analysis and calculated by the $2^{-\Delta Ct}$ method. Columns in red show the FPKM values of genes obtained by RNA-seq analysis. Values are mean ± SD of four (n = 4) and three (n = 3) replicates derived by qRT-PCR and RNA-seq analysis, respectively. Asterisks indicate significant differences (*t*-test) between the *Cmm* infected and control plants ($P \leq 0.05$).

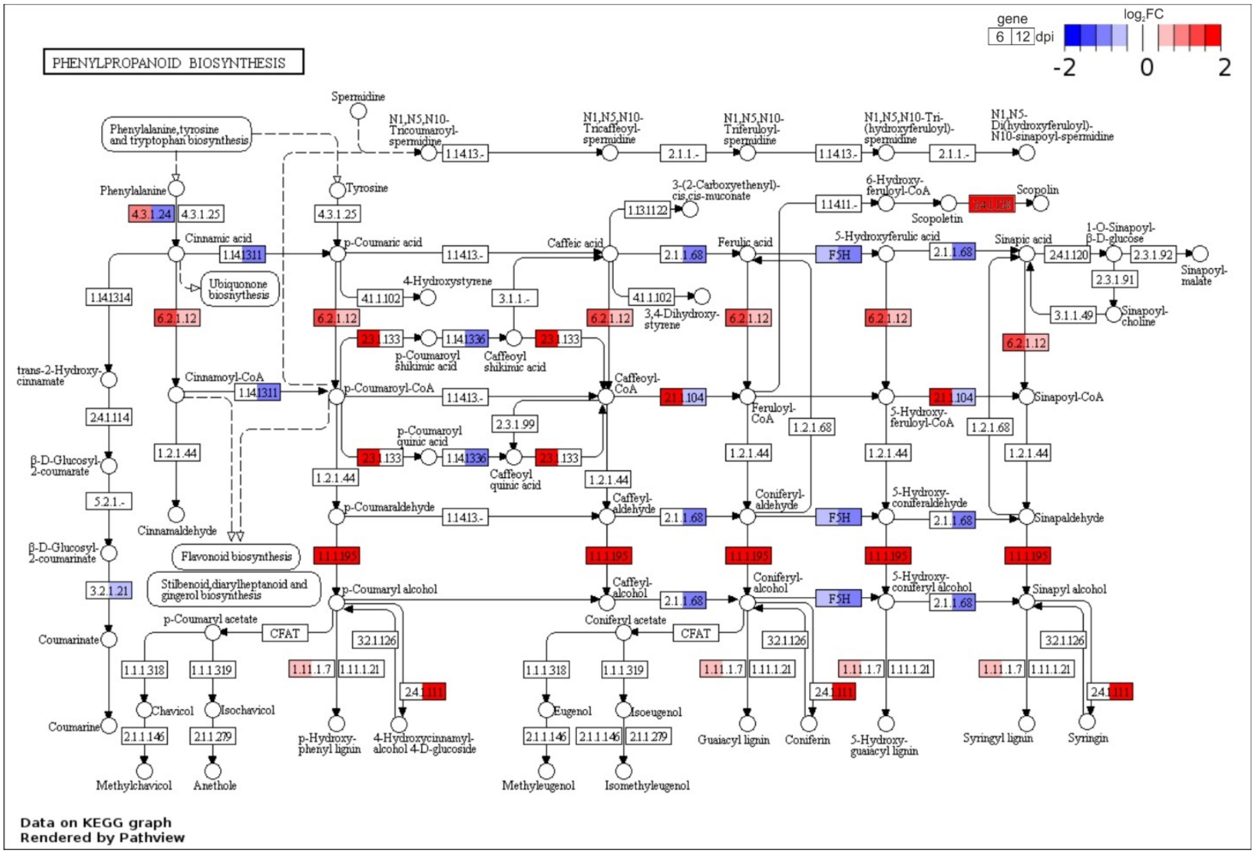

Supplementary Figure S6. Pathview graph of the KEGG pathway of phenylpropanoids metabolism at 6 and 12 dpi.
